# The Clinical Utility of Measuring IgG Subclass Immunoglobulins During Immunological Investigation for Suspected Primary Antibody Deficiencies

**DOI:** 10.1093/labmed/lmx058

**Published:** 2017-11-08

**Authors:** Antony R Parker, Markus Skold, David B Ramsden, J Gonzalo Ocejo-Vinyals, Marcos López-Hoyos, Stephen Harding

**Affiliations:** 1 The Binding Site Group Limited, Edgbaston, Birmingham;; 2 Institute of Metabolism and Systems Research, The Medical School, University of Birmingham, Birmingham, UK;; 3 Immunology Department, Hospital Universitario Marqués de Valdecilla-IDIVAL, Santander, Spain.

**Keywords:** IgG subclass, low IgA, specific antibody deficiency, common variable immunodeficiency

## Abstract

Measurement of IgG subclass concentrations is a standard laboratory test run as part of a panel to investigate the suspicion of antibody deficiency. The assessment is clinically important when total IgG is within the normal age-specific reference range. The measurement is useful for diagnosis of IgG subclass deficiency, to aid the diagnosis of specific antibody deficiency, as a supporting test for the diagnosis of common variable immunodeficiency, as well as for risk stratification of patients with low IgA. The measurement of IgG subclasses may also help determine a revaccination strategy for patients and support patient management. In certain circumstances, the measurement of IgG subclasses may be used to monitor a patient’s humoral immune system. In this review, we discuss the utility of measuring IgG subclass concentrations.

Differentiating individuals with recurrent infections (RIs) because of a compromised immune system from those whose immune systems operate at the edge of normal function may be challenging, particularly in the case of pediatric age groups. Normal pediatric age groups may have many respiratory infections in any one year,^1-3^ and additionally, those individuals whose transient immune deficiency will resolve over time must be distinguished from those whose deficiency is more permanent. To resolve the above issues, detailed immunological analyses may help to ensure early diagnosis and appropriate treatment. This is imperative because of the high risk of RI, which may cause irreversible organ damage. 

The measurement of IgG subclass antibodies (IgGSc) is included in a number of protocols and guidelines for investigating individuals presenting with RIs, lung diseases, and strong suspicion of an antibody deficiency (Table 1). These analyses are also helpful in 1) monitoring of transient versus permanent antibody deficiencies, 2) assessing the progression of mild immunodeficiencies into more severe forms, and 3) supporting some treatment decisions (summarized in Table 2).

**Table 1.** Protocols and Guidelines Documenting the Measurement of IgGSc in Immunological Investigation in the Last 10 Years

Reference	Title	Recommendation for IgGSc Measurement
Slatter and Gennery ^111^	Clinical immunology review series: an approach to the patient with recurrent infections in childhood.	IgGSc testing as firstline testing alongside IgG, IgA and IgM
De Vries et al^16^	Patient-centred screening for primary immunodeficiency, a multi-stage diagnostic protocol designed for non-immunologists: 2011 update.	IgGSc testing after IgG, IgA and IgM testing
Chang et al^12^	Chronic suppurative lung disease and bronchiectasis in children and adults in Australia and New Zealand.	IgGSc testing as first-line testing alongside IgG, IgA and IgM
Ameratunga et al^83^	New diagnostic criteria for common variable immune deficiency (CVID), which may assist with decisions to treat with intravenous or subcutaneous immunoglobulin	Measurement of IgG3 as supportive laboratory evidence
Ameratunga et al^84^	New diagnostic criteria for CVID	Measurement of IgG3 as supportive laboratory evidence
Ladomenou and Gaspar^19^	How to use immunoglobulin concentrations in investigating immune deficiencies	IgGSc testing after IgG, IgA, and IgM testing

Older protocols and guidelines include the following:

De Vries et al^14^,

^17^

Cunningham Rundles^13^

Folds and Schmitz^18^

**Table 2.** Summary of the Clinical Utilities of IgGSc Measurement

Utility of IgGSc Measurements
Diagnosis
1. Isolated IgGScD
2. Specific antibody deficiency
3. Clinically relevant IgGScD
4. Transient hypogammaglobulinemia of infancy
Supporting information/risk of infection
1. Patients with low IgA
2. Common variable immunodeficiency
Monitoring
1. Transient hypogammaglobulinemia of infancy/permanent antibody deficiency
2. Progression to a more complex antibody deficiency
Treatment
1. Support treatment decisions (revaccination, antibiotics, antibody replacement therapy, etc)

IgGSc deficiency (IgGScD) is clinically and genetically heterogeneous, and may coexist with abnormal expression of more than 1 subclass antibody. Individuals with IgGScD may present with a combination of frequent or severe bacterial infections of the upper and lower respiratory tract,^4-6^ allergic asthma and allergic rhinitis,^7,8^ and autoimmune conditions.^7,9-11^

In this review, we discuss the clinical situations in which measurement of IgGSc has been reported to be of benefit.

## Diagnosis of IgGSc Deficiency

The measurement of IgGSc has been employed as a screening tool for individuals presenting with multiple chronic illnesses or RI (Table 1). Some authors have reported measuring IgGSc as a frontline test alongside the measurement of total IgG, IgA, and IgM,^12-15^ but more commonly, the measurement of IgGSc is a second-line test.^16-19^ De Vries^16^ has suggested that IgGSc are measured after the measurement of IgG, IgA, and IgM usually if IgG is higher than 4 g/L and alongside the response to vaccination. Ladomenou and Gaspar^19^ have suggested that IgGSc concentrations are measured after the assessment of the response to vaccines. Further, IgGSc are useful as part of an evaluation in studying patients with a strong history of infections, normal major isotypes, and normal antibody responses. As discussed below, IgGSc are also recommended as second-line tests in patients with low IgA and should be monitored in IgGScD patients for progression to common variable immunodeficiency (CVID).^20-25^ Whether first or second line, the measurement of IgGSc is an important test that provides a more complete picture of the humoral immune system.

Concentrations of IgGSc are commonly measured using radial immunodiffusion (RID), enzyme-linked immunosorbent assay (ELISA), and the more automated methods of nephelometry and turibidimetry. All methods provide quantitative measurement of the IgGSc concentrations, and all assays provide a good level of sensitivity for detection of low IgGSc concentrations. RID is commonly used if the testing numbers are low, and nephelometric and turbidimetric assays are operated on automated high throughput analysers on which other laboratory assays are run. ELISA may use monoclonal antibodies for detection rather than the polyclonal antibodies used in RID and nephelometric or turbidimetric assays. Time to first result is slower with RID due to incubation times required for antigen-antibody ring formation. For nephelometric and turbidimetric assays, differences exists between manufacturers’ assays.^26-29^

Reference ranges for pediatric age groups and adult populations have been developed^30,31^ (Table 3), as well as some study-specific ranges.^7,32,33^ The diagnosis of IgGScD requires one or more IgGSc concentrations to be less than the fifth percentile in the presence of normal concentrations of IgG, IgA, and IgM.^34^ In patients older than 15 years of age, it has been reported the median IgG3 concentration in patients with IgG3 subclass antibodies deficiency (IgG3ScD) is approximately 0.19 g/L (range 0.10-0.34) and less than 0.10 to 0.24 g/L in patients with chronic sinusitis, and that IgG2 concentrations were less than 0.25 g/L in patients with low IgA and IgG2 subclass antibodies deficiency (IgG2ScD).^7,32,33^ All abnormal IgGSc concentrations should be confirmed with at least one additional measurement one month after the first test.^34,35^ IgGScD may be due to abnormal concentrations of one IgGSc only or different combinations of any of the four IgGSc.

**Table 3.** Mean (50th Percentile) IgGSc Concentration and Reference Intervals (2.5-97.5 Percentiles) With Respect to Age Data obtained from Schauer et al^31^

		Age Range									
		0.5-1 year	1-1.5 years	1.5-2 years	2-3 years	3-4 years	4-6 years	6-9 years	9-12 years	12-18 years	Adult
IgG1 (g/L)	Mean	2.9	3.5	4.0	4.5	4.8	5.0	5.7	6.0	5.8	5.0
	Reference interval	1.4-6.2	1.7-6.5	2.2-7.2	2.4-7.8	2.7-8.1	3.0-8.4	3.5-9.1	3.7-9.3	3.7-9.1	2.8-8.0
IgG2 (g/L)	Mean	0.58	0.62	0.80	0.95	1.15	1.30	1.70	2.10	2.60	3.0
	Reference interval	0.41-1.30	0.4-1.40	0.5-1.80	0.55-2.00	0.65-2.20	0.7-2.55	0.85-3.30	1.0-4.00	1.1-4.85	1.15-5.70
IgG3 (g/L)	Mean	0.41	0.42	0.44	0.46	0.48	0.50	0.54	0.58	0.63	0.64
	Reference interval	0.11-0.85	0.12-0.87	0.14-0.91	0.15-0.93	0.16-0.96	0.17-0.97	0.20-1.04	0.22-1.09	0.24-1.16	0.24-1.25
IgG4 (g/L)	Mean	0.002	0.030	0.068	0.138	0.201	0.257	0.368	0.469	0.491	0.349
	Reference interval	0.000- 0.008	0.000- 0.255	0.000- 0.408	0.006- 0.689	0.012- 0.938	0.017- 1.157	0.030- 1.577	0.043- 1.900	0.052- 1.961	0.052-1.250

Knowledge of IgGSc concentrations is clinically important when total IgG, IgA, and IgM concentrations are within their normal ranges,^17,19,33^ and in individuals with RI, a high percentage may have normal IgG concentrations (Table 4).

**Table 4.** Examples of the Measurement of IgG and IgGSc for Screening Individuals Presenting With Different RIs

Type of Infection	Normal IgG (%)	Low IgGSc Concentration	Comments Regarding IgGScD	Reference
Chronic and recurrent ear, nose, and throat	101/103 (98)	4/101 (4)	Response to Pneumococcal vaccination normal in 1/4 IgGScD (25%) 1/4 IgGScD developed bronchiectasis	Aghamohammadi et al^78^
Upper respiratory tract infection with otitis media/lower respiratory tract infection with pneumonia	22/55 (47)^a^	22/22 (100)	IgGScD as follows: 3 IgG1, 8 IgG2, 11 IgG3. 3/7 patients with low IgA had IgGScD.	Bossuyt et al ^71^
Recurrent bronchitis	23/25 (92)	17/25 (68)	IgGScD as follows: 2 IgG2, 4 IgG3 and 7 IgG4. 4 combined IgGScD.	De Baets et al^55^
Chronic and recurrent rhinosinusitis	70/74 (95)	33/70 (47)	5 low IgG1, 3 low IgG2, 19 low IgG3, 6 low IgG4—7 with >1 low IgGSc	Scadding et al^33^
Therapy refractory recurrent rhinosinusitis	240/245 (98)	17/240 (7)	IgGScD as follows: 5 IgG1, 10 IgG2, 1 IgG3, and 1 IgG4. 3/17 had an inadequate pneumovax response	May et al^112^

^a^In 2 patients with low IgG2, they had low total IgG.

## Mechanism of IgGScD

The human immunoglobulin heavy chain constant region (*IGHC*) is localized on chromosome 14 and contains 9 functional genes *(μ-δ-γ3-γ1-Ψε-α1-Ψγ-γ2-γ4-ε-α2*).^36^ The frequency of *IGHC* gene deletions in the Caucasian population is approximately 1.5%. The linkage pattern dictates that many of the deletions occur in discrete blocks such as IgG1 and IgG3, IgG2 and IgG4, and IgA1, IgG2, and IgG4, which may be extended to include IgG1 and IgE. *IGHC* gene deletions have been reported in the normal healthy population, calling into question the clinical relevance of IgGScD.^36-41^

Plebani et al^41^ reported 2 siblings that had IgGScD with undetectable serum concentrations of IgA1, IgG2, IgG4, and IgE due to a homozygous deletion. Serum IgG, IgG1, and IgG3 concentrations were higher than expected in normal individuals, and they displayed a normal IgG response to tetanus toxoid and polysaccharide antigens with increased IgG1 and IgG3 isotypes. Similarly Hammarstrom et al^42^ reported IgG3 and IgG4 compensation in an individual with IgG1 subclass antibody deficiency (IgG1ScD), and a shift in the isotype of antipolysaccharide antibodies to IgG1 and IgG3 in IgG2ScD individuals. The high level of homology of the *IGHC* gene favors unequal crossing-over events, and the compensatory mechanisms reported for the gene deletions may explain the lack of significant infections in the healthy population and thus suggest these gene deletions are nonpathogenic.

Immunoglobulin deficiencies are mainly caused by immunoregulatory dysfunctions such as aberrations in 5’ regulatory sequences, in 3’ regulatory sequences, or in the production or response to selected cytokines (reviewed in Pan and Hammarstrom^36^) are probably the cause of the more pathogenic IgGScD.

There may be prognostic importance in distinguishing between IgGScD caused by gene deletion and that due to immunoregulatory dysfunction as those with the latter may have the higher risk of RI.

## IgGScD

**Figure 1:**
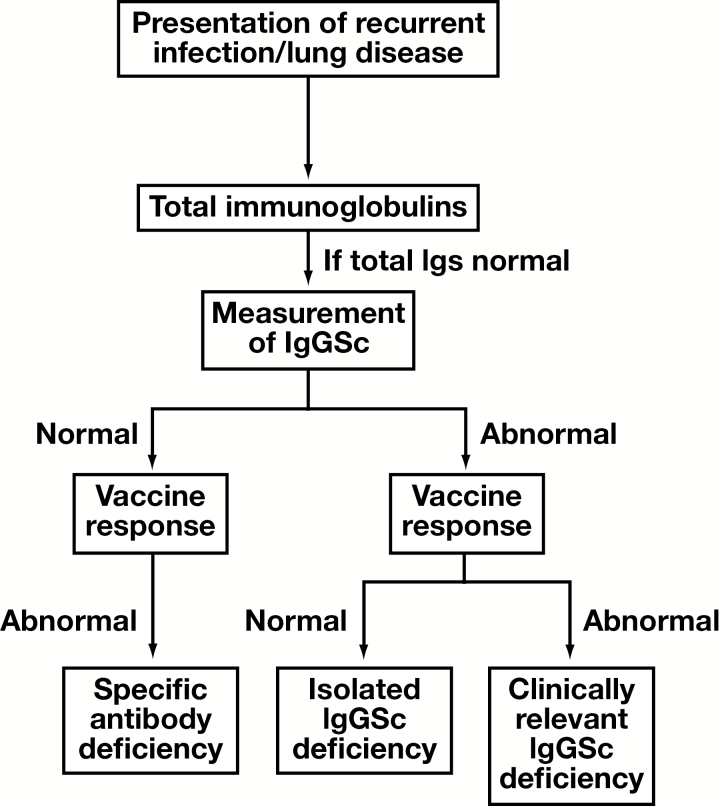
Algorithm for the measurement of IgGSc in individuals with suspicion of antibody deficiency.

IgGScD is one of the most common primary antibody deficiencies (PADs) in both pediatric age groups and adults presenting with RI (Figure 1 and Table 4).^43,44^ The most common clinical associations of IgGScD are recurrent encapsulated bacterial and viral respiratory tract infections.^7,45-47^ The frequency of recurrent pneumonia is significantly higher in IgGScD than that observed in the normal population. Ozkan et al found that the rate of chronic pulmonary damage was 5 times higher in pediatric age groups with IgGScD than those with low IgA.^46^ It has been reported that bronchiectasis may already be present in as many as 10% of IgGScD pediatric patients at diagnosis.^43^ In a study of 350 adult IgGScD patients, 29% had asthma, 9% had asthma combined with chronic obstructive pulmonary disease (COPD), and 9% had COPD alone.^48^

IgG2- and IgG3- are the most common IgGScDs,^7,43,45-47^ presumably in part because IgG1 deficiency can closely mirror IgG deficiency. IgG2ScD may be associated with some restricted responses to polysaccharide antigens, and in IgG3ScD, the response to *Moraxella catarrhalis*, *Streptococcus pyogenes,* and respiratory syncytial virus infection may be compromised.^49,50^ IgG4 is present at very low concentrations in healthy individuals; thus IgG4 subclass antibodies deficiency (IgG4ScD) may be difficult to diagnose. IgG2ScD is sometimes accompanied by low IgG4 concentrations,^34^ and a deficiency of IgG1 is more likely associated with low IgG3 concentrations. IgGScD has been observed as a comorbidity in other primary immunodeficiencies (PIDs), such as ataxia telangiectasia,^51,52^ and secondary immunodeficiencies, such as chronic lymphocytic leukemia.^53^

Some abnormalities of specific polysaccharide antibody production have been described in patients with IgG1ScD, IgG2ScD, and IgG3ScD.^54-58^ IgG2ScD patients have antibody responses to a restricted number of polysaccharides in the Pneumovax polysaccharide vaccine^56,59,60^ and the association between decreased IgG3 concentrations and lack of pneumovax response have been reported.^61^ De Gracia and colleagues reported that the prevaccination, postvaccination concentrations as well as fold-increase in antibody concentrations in response to the *Haemophilus* conjugate vaccine were significantly lower in individuals with IgGScD.^62^ Defective response to vaccination needs to be considered in cases of suspected IgGScD, as this may indicate a more severe antibody deficiency (clinically relevant IgGScD) rather than an isolated IgGScD.^47^ Likewise, normal IgGSc with defective response to vaccination may indicate a specific antibody deficiency (SAD, summarized in Figure 1). Along with the clinical presentation, these distinctions may support a reliable diagnosis and treatment regime.

## Measurement of IgGSc in Other Antibody Deficiencies

### IgGScD in Patients with Low IgA

The majority of patients with low IgA are asymptomatic, but approximately 30% present with infection, eg recurrent viral infections, recurrent otitis media, frequent sinopulmonary infections, or gastrointestinal infections.^63^ In addition, low IgA has been associated with abnormalities of antibody-mediated immunity, such as IgE deficiency,^64^ deficient serum IgGSc concentrations,^65-68^ and impaired antibody responses against both protein and polysaccharide antigens.^65,69,70^ In pediatric age groups presenting with RI, a percentage may have an IgGScD dependent on presentation or type of RI.^21,55,71^ The additional defective ability to produce normal IgGSc in the background of low IgA may lead to more frequent and more severe infections than those present in patients with normal IgA^20-25,68^ and is readily observed in patients who present with recurrent bronchitis.^22,23,68,72-76^ When patients with low IgA were grouped according to severity of infection (none, mild, and severe), although the IgA concentrations were not significantly different, mean IgG4 concentrations were lower in the group with mild infections and even lower in the group with severe infections compared to the group with no infections.^65^ A higher frequency of infection has also been reported in patients with low IgA and IgG2ScD compared to those with low IgA alone.^32,77^

**Figure 2:**
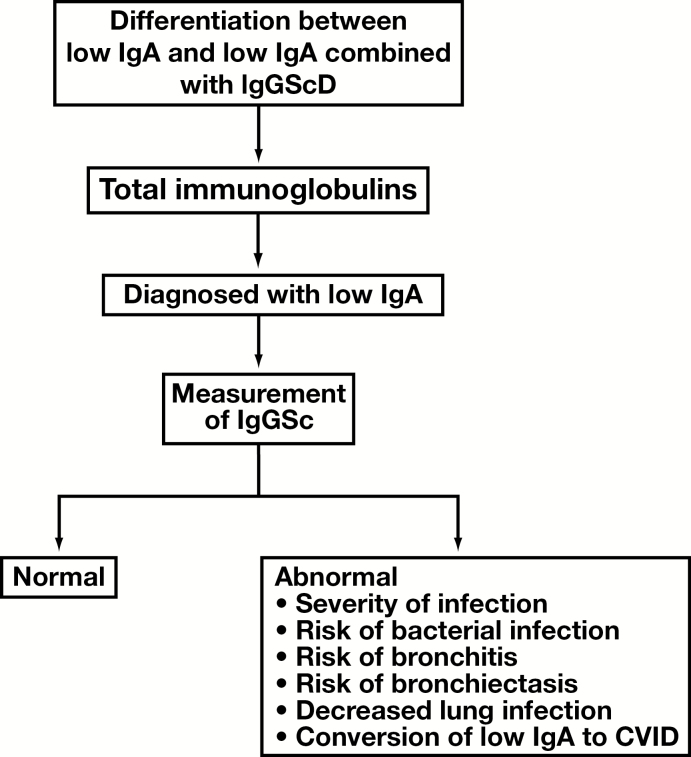
Algorithm for the measurement of IgGSc in individuals with low IgA.

The high rates of infection in patients with low IgA and IgGScD result in decreased lung function,^72^ and in particular IgG4ScD correlates with the presentation of bronchiectasis.^78^ In some cases, low IgA may evolve into the more severe CVID, and a decrease in IgGSc concentrations may occur before a decrease in total IgG.^20,78,79^ An algorithm for the testing of IgGSc in individuals with low IgA and utility is shown in Figure 2.

Finally, and although it is outside the scope of this review, patients with low IgA may present with antibody-mediated autoimmune diseases that reflect a dysregulation of the humoral response.^80^ However, little evidence is found in the literature about the association of low IgA and IgGScD with autoimmunity.

### IgGScD in CVID Patients

CVID is the most frequent clinically symptomatic primary antibody disorder in adults with a marked decrease of IgG and at least 1 of IgM or IgA. The prevalence is approximately 1:25,000 to 1:50,000, and in most patients the genetic cause remains undefined.^81,82^ The onset of CVID occurs after 4 years of age. Diagnosis requires prior exclusion of hypogammaglobulinemia and that patients have a poor response to vaccines.^34,81^ Ameratunga and colleagues have proposed updated criteria for the diagnosis of CVID, based on recent evaluation of the European Society of Immune Deficiencies (ESID) and the Pan American Group for Immune Deficiency (PAGID) definitions of CVID.^83,84^ The diagnostic algorithm requires sequential steps.

**Figure 3:**
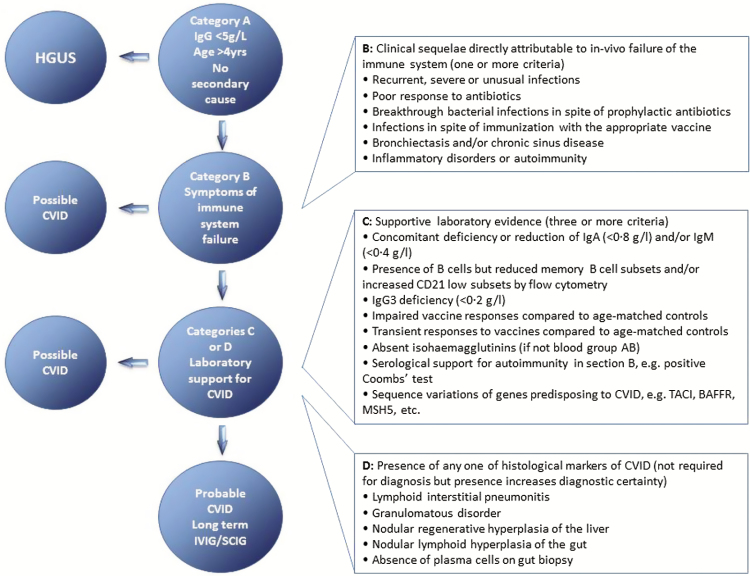
Diagnostic and treatment algorithm for patients with hypogammaglobulinaemia and CVID. Data obtained from Ameratunga et al.^83^

The evaluations in category A are required to consider a diagnosis of CVID, and to differentiate between those with mild hypogammaglobulinemia of uncertain significance (HGUS) and those whose presentation is more suggestive of CVID (Figure 3). Category B details the clinical sequelae from the in vivo failure of the immune system. The determination of recurrent, severe, or unusual infections, particularly in the presence of prophylaxis and treatment, is important, as is excluding infection due to anatomical and functional defects. Category C is the provision of serological and genetic data to support the suggestion of CVID. Many CVID patients have a deficiency of IgG, IgA, and/or IgM, and many have reduced memory B cell subsets. The response to vaccination and concentration of isohemagglutinins are important to assess, as are the serological indications for autoimmunity. Determination of sequence variations in genes known for predisposing one to CVID is important. IgG3ScD may be considered a supportive marker for a defective immune system and should be given similar status to impaired memory B cells. The significance of reduction of any other IgGSc is currently unknown. The final category, D, is the identification of lesions that may be associated with CVID.

Patients with probable CVID must fulfill all criteria in category A, at least 1 from category B, and 3 or more from category C. Category D does not need to be fulfilled, but strengthens the diagnosis of CVID. For patients who do not fulfill category A, one may consider HGUS. Many patients may not fulfill categories B and/or C, and may have possible rather than probable CVID. Some CVID patients present with severe hypogammaglobulinemia and significant autoimmunity, rather than an increased susceptibility to infections. These patients may have a diagnosis of CVID if they meet the criteria in category A as well as those in categories C or D.

The recent International Consensus Document (ICON) for common variable immunodeficiency disorders suggests that the diagnosis of CVID does not require the additional laboratory criteria reported in Ameratunga et al.^83,84^ Bonilla et al suggest that milder laboratory phenotypes such as IgGScD may evolve over time into CVID, and so monitoring of IgGScD patients may be applicable for early detection of any transformation.^81^

## Measurement of IgGSc Concentrations to Aid the Diagnosis of Specific Antibody Deficiency

It is important to differentiate between isolated IgGScD, SAD, and clinically relevant IgGScD, since the decision process for treatment and management may differ. Individuals with SAD differ from those with isolated IgGScD and clinically relevant IgGScD, since they have normal IgGSc concentrations and always have an impaired response to pneumococcal polysaccharide vaccination.^34,85^ Patients with SAD are predisposed to sinopulmonary bacterial infections, sinusitis, otitis, bronchitis, and pneumonia with pathogens, including *Streptococcus pneumoniae*, *Moraxella catarrhalis*, *Haemophilus influenzae*, and *Streptococcus aureus.*

Patients with SAD are managed in a variety of ways, including vaccination with conjugated vaccines, prophylactic antibiotics, and occasionally with antibody replacement therapy. This may differ from the patient management employed for those with isolated IgGScD and certainly for those with clinically relevant IgGScD with respect to antibody replacement. This may be indicative of the differing severity of immune defect among these PIDs (Figure 3).

Initial treatment of patients with normal IgG but IgGScD or SAD may include antibiotics for the infections and vaccination with the pneumococcal conjugate vaccine.^86^ A clear purpose for antibody replacement therapy is reduced levels of serum IgG (<2 g/L) in patients with recurrent bacterial infections, but this may be more difficult to justify in the presence of normal total IgG. The justification may be strengthened, however, for patients with clinically relevant IgGScD, as both IgGSc concentrations and response to vaccination are compromised. Antibody replacement therapy may be indicated in SAD patients, or patients with isolated IgGScD or clinically relevant IgGScD if the patients present with severe and/or RI,^87^ even though prophylactic antibiotics have been administered.^48^ The decision-making process is governed by clinical presentation, may involve the patients response to treatment, and is a clinician-dependent justification. Measurement of IgGSc is required to support the clinical presentations and the decisions for appropriate treatment.^87,88^

## Measurement of IgGSc as a Monitoring Tool

### Differentiation Between Transient and Persistent Antibody Deficiencies

A challenging issue for the clinician is whether the clinical symptoms presented are the result of a slow maturing immune system or an underlying immune deficiency. Transient hypogammaglobulinemia of infancy (THI) is an antibody deficiency occurring in the first years of life that is characterized by a delay in immunoglobulin production that spontaneously recovers in early infancy. Only those patients whose clinical symptoms have resolved and IgG concentrations have normalized after the age of 4 years have definitive THI. The diagnosis is made a posteriori.^34,89^

Routine measurement and normalisation of IgGSc may differentiate those who have a transient immunodeficiency from those who may have a more persistent polyclonal immunodeficiency.^43,90-94^

During the follow-up of 24 pediatric age group patients with IgGScD over 40 months, 25% had no further RIs, which correlated with IgGSc normalisation in all individuals tested.^43^ Kutukculer and colleagues reported that the IgGSc concentrations in 67% of pediatric age group patients with low IgA and IgGScD and 30% of pediatric age group patients with isolated IgGScD will normalize to age-specific normal concentrations.^95^ It has been reported that the mean age for normalization of IgGSc concentrations in pediatric age group patients was between 4 and 6 years of age,^91,96^ and that low IgG2 concentrations may be more challenging to normalize, signifying a more permanent antibody deficiency.^91,92^

The measurement of IgGSc may serve as suitable diagnostic tool for THI to support the change in presentation that accompanies IgGSc normalization. Moschese and colleagues reported the normalization of IgG in 41/57 children with hypogammaglobulinemia thus suggesting the diagnosis of THI.^89^ Of these 41 children, 18 (44%) still had episodes of infection, 61% with upper respiratory tract infections and 5.5% with pneumonia. The indication of infections in individuals with THI and normal IgG may warrant the measurement of IgGSc.^47,89,97^ Stiehm et al has suggested that patients diagnosed with THI may develop IgGScD.^47^

**Figure 4:**
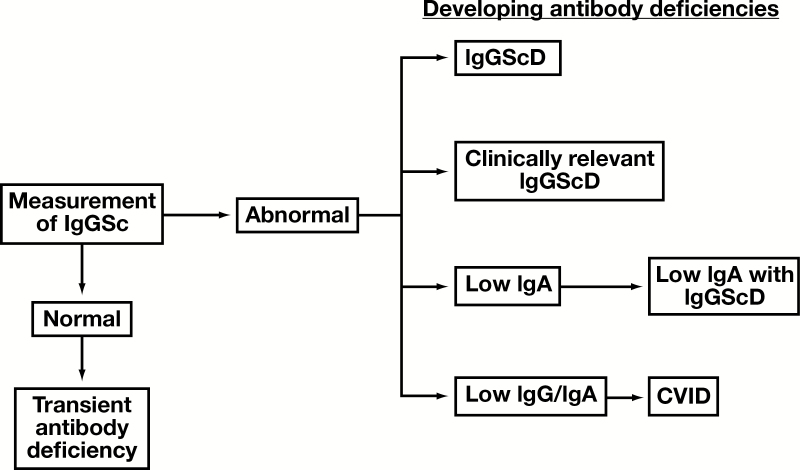
Summary for measuring IgGSc concentrations as a monitoring tool. Normalization of IgGSc may support the diagnosis of THI and exclude suspicion of a more permanent antibody deficiency. The failure of IgGSc concentrations to normalize may be indicative of a more permanent IgGScD or the evolution of a more severe antibody deficiency. The antibody defect may remain IgGScD or develop further into other deficiencies. Examples may include accompanying a progressive deficient response to vaccines developing into clinically relevant IgGScD, development of low IgA alone with IgGScD, or the further development of low IgG or IgA in the progression to CVID.

In addition to normalization of IgGSc suggesting a transient rather than permanent antibody deficiency, failure of the IgGSc concentrations to normalize may be a first step in identifying an underlying progressive immunodeficiency (Figure 4). In pediatric age group IgGScD patients, 11/24 (46%; >10 years of age) progressed to a more severe antibody deficiency; 1 developed low IgA, 3 developed IgG deficiency (IgGD), 3 developed IgGD with low IgA, and 4 developed CVID.^43^ Further reports have suggested that a IgGScD may develop after diagnosis of low IgA and correlate with severity of infection, and that the IgGScD may precede IgGD and gradual development of CVID.^20,98-100^ De Vries and colleagues have recommended that pediatric age groups with IgGScD should have their IgGSc concentrations measured every 1 to 2 years.^14,101^ ICON guidelines suggest that milder laboratory phenotypes such as IgGScD may evolve over time into more severe antibody deficiencies, and so monitoring of IgGScD patients may be applicable for early detection of any transformation.

## Influence on Treatment Regimes

### Immunoglobulin Replacement Therapy

Current guidelines for the treatment of IgGScD suggest treating any allergic symptoms and support the administration of prophylactic antibiotics. In selected patients, cautious use of polyclonal human IgG may be required.^87,88^

Immunoglobulin prophylaxis reduces the frequency of RI in IgGScD patients^5,48,102-105^ and those with low IgA and low IgG2.^72,106,107^ Genel and colleagues used a prophylactic regime (oral immunomodulator bacterial extract OM-85 BV or benzathine penicillin) for patients with IgGScD alone and low IgA with IgGScD.^102^ At 12 months, the number of infections and antibiotic courses decreased significantly. In IgGScD patients receiving a prophylactic regime of antibiotic prophylaxis, oral bacterial lysate, and/or intravenous immunoglobulin, the frequency of RI decreased.^95,97^ Measurement of IgGSc may further identify individuals who may benefit from the appropriate treatment.

### Revaccination

Reports suggest some correlation between IgG2ScD and the defective antibody response to polysaccharide antigens.^24,56^ Vaccination with the conjugated pneumococcal polysaccharide vaccine, however, has been shown to be effective in patients with IgG2ScD, since the response is directed towards the protein conjugated immunogen rather than the polysaccharide backbone.^86,108^ Identification of an IgG2ScD may illicit revaccination with the protein conjugated vaccine. The ICON study recommends impaired vaccine antibody responses for a diagnosis of CVID,^81^ while Ameratunga and colleagues suggest that the response to vaccination is not an important part of the diagnostic criteria.^83,84^ Goldacker and colleagues supported this further by reporting the response to vaccines in some CVID patients.^109^ Measurement of IgGSc may be of interest to determine who requires revaccination or further vaccination, and indeed whether to proceed with polysaccharide or conjugated polysaccharide vaccination.^86,110^

## Conclusions

The measurement of IgGSc is a frequently requested laboratory test whose extensive clinical indication requires complete understanding.

Individuals are often referred to immunologists for the evaluation of reduced serum immunoglobulins. Normal values of IgG, IgA, and IgM are not always enough to exclude a more serious condition, and alongside the information obtained from measurement of specific antibody production and B lymphocyte populations, measurement of IgGSc can provide a more complete view of the function of the humoral immune system.^19^

Measurement of IgGSc may occur as a frontline test alongside the measurement of total immunoglobulins,^12-15^ but more commonly, the measurement of IgGSc is a second-line test^16-19^ given alongside or after the response to vaccines. Reference ranges for pediatric age groups and adult populations have been developed to aid identification of low concentrations, and a minimum of 2 measurements are required to confirm a diagnosis of IgGScD. Several methods are available for quantitation of the IgGSc concentrations, and several differences exist between manufacturers’ assays within a certain technique. This may influence quantitation, and therefore care must be taken to ensure serial monitoring for patients is performed within a single laboratory rather than across multiple laboratories to ensure a single technique is used.

Measurement of IgGSc can support the diagnosis of CVID and aid in the risk stratification of individuals with low IgA. The utility of the IgGSc is most prominent when combined with the response to vaccination. The discrimination between isolated IgGScD, SAD, and clinically relevant IgGScD is particularly important since this may influence treatments. There is clear utility for the measurement of IgGSc as a monitoring tool. The use alongside the measurement of total IgG may further aid differentiation between THI and a more permanent PAD. Prognostic information may be obtained by defining the cause of the deficiency.

IgGScD may influence frequency and severity of infection. The measurement of IgGSc should always be considered within a panel of other tests. In PADs the measurement of IgGSc and the response to vaccination provides a comprehensive window into the function of the immune system. LM

### Disclosures

Antony R Parker, PhD, Markus Skold, PhD, and Stephen Harding, PhD, are employees of the Binding Site Group Limited, which manufactures IgG subclass assays.

## Abbreviations

COPD: chronic obstructive pulmonary disease
CVID: common variable immunodeficiency
ELISA: enzyme-linked immunosorbent assay
ESID: European Society of Immune Deficiencies
HGUS: hypogammaglobulinemia of uncertain significance
ICON: International Consensus Document
IgGD: IgG deficiency
IgGSc: IgG subclass antibodies
IgGScD: IgGSc deficiency
IGHC: immunoglobulin heavy chain constant region
PAD: primary antibody deficiency
PAGID: Pan American Group for Immune Deficiency
PID: primary immunodeficiency
RI: recurrent infection
RID: radial immunodiffusion
SAD: specific antibody deficiency
THI: transient hypogammaglobulinemia of infancy.
